# Optimizing intubation technique using a single-use video laryngoscope: A comparative study in a simulation model

**DOI:** 10.1097/MD.0000000000038946

**Published:** 2024-07-12

**Authors:** Hui-Chin Chen, Jui-Fang Liu, Miao-Ching Chi, Hsiu-Lan Cheng

**Affiliations:** a Department of Respiratory Care, Chang Gung University of Science and Technology, Puzi City, Taiwan, ROC; b Chronic Diseases and Health Promotion Research Center, Chang Gung University of Science and Technology, Puzi City, Taiwan, ROC; c Division of Pulmonary and Critical Care Medicine, Chiayi Chang Gung Memorial Hospital, Puzi City, Taiwan, ROC; d Department of Adult and Continuing Education, National Chung Cheng University, Puzi City, Taiwan, ROC.

**Keywords:** conventional laryngoscope, COVID-19, intubation techniques, video laryngoscope

## Abstract

Tracheal intubation poses a high risk of infection to medical staff due to Coronavirus disease 2019 (COVID-19) highly infectious nature. To mitigate this risk, various medical devices, including video laryngoscopy, have been developed to assist intubation. This study compared conventional laryngoscopy (Macintosh) and disposable video laryngoscopes (Medcaptain VS-10s and Honestmc Laryngoscope_LA10000) in terms of their use and operation processes. We designed a questionnaire to assess the operator perception of performing intubation with the devices, and statistical analysis was performed on 50 clinical staff members from 2 hospitals who had performed intubation or had learned intubation techniques. The primary outcomes were time to glottic visualization, intubation time, intubation success rate, distance between the operator and training model, and time from glottic visualization to tube insertion. The secondary outcomes were as follows: overall laryngoscope quality, operative feel, maneuverability, ease of use, and video quality. This study showed that video laryngoscopes were superior to conventional laryngoscopes in terms of quality, operative feel, and ease of use. When LA10000 was employed, the intubation success rate was higher, and the operator risk of infection was lower because of the greater distance from the training model. However, the use of video laryngoscopes requires appropriate education and training use of the devices. This study also demonstrated that when participants viewed a simple operation video prior to using video laryngoscopes, tube insertion time was shorter. Overall, video laryngoscopy can provide a safer and more convenient option for clinical medical personnel during pandemics.

## 1. Introduction

Endotracheal intubation is an effective method for maintaining airway patency and is a common operation in emergency rescue^[1]^ and intensive care.^[2]^ The difficult part of endotracheal intubation is exposure of the glottis. When intubation is difficult, multiple trial insertions and catheterization of the throat can cause severe cardiovascular reactions in patients, affecting their treatment and threatening their lives. The hemodynamic response during laryngoscopy and intubation depends directly on the force applied and duration of intubation.^[3,4]^ Various auxiliary intubation medical devices have been developed to solve the problem of failure of tracheal intubation caused by glottic exposure, including video laryngoscopes.

The aim of video laryngoscopy is to improve the success rate of intubation and reduce anesthesia-related morbidity and mortality.^[5]^ A video laryngoscope is a medical device used to visualize the larynx during intubation. It consists of a camera, display, and blade with a light source. Conventional intubation techniques do not involve the use of a video laryngoscope. Instead, techniques such as direct laryngoscopy, indirect laryngoscopy, and fiberoptic intubation are used. Video laryngoscopy is superior to conventional laryngoscopy, offering a superior laryngeal view, and resulting in a higher intubation success rate.^[6–9]^

Droplet transmission of the highly contagious Coronavirus disease 2019 (COVID-19) is a major risk for intubation personnel because the intubation process can easily result in aerosol scattering, leading to a high risk of infection to medical staff performing intubation. Therefore, a light and easy-to-carry video laryngoscope with a disposable blade was designed. As relevant products on the market have limitations. Firstly, they come with a significant learning curve, requiring operators to undergo training to use them proficiently. The cost of these devices is often high, posing a financial burden for resource-limited healthcare facilities. Additionally, regular maintenance and calibration are necessary to ensure proper functionality, which adds to the complexity and expense of their use. Lastly, while there are single-use options, reusable equipment necessitates stringent disinfection processes to prevent cross-contamination. These factors highlight the challenges in the practical application of modern intubation devices despite their advantages.

This study compared the use and operation of a conventional laryngoscope (Macintosh) with those of disposable video laryngoscopes (Medcaptain VS-10s & Honestmc Laryngoscope_LA10000). In this study, disposable video laryngoscopes refer to blades intended for single use only.

## 2. Methods

### 2.1. Research design

**Figure 1.**
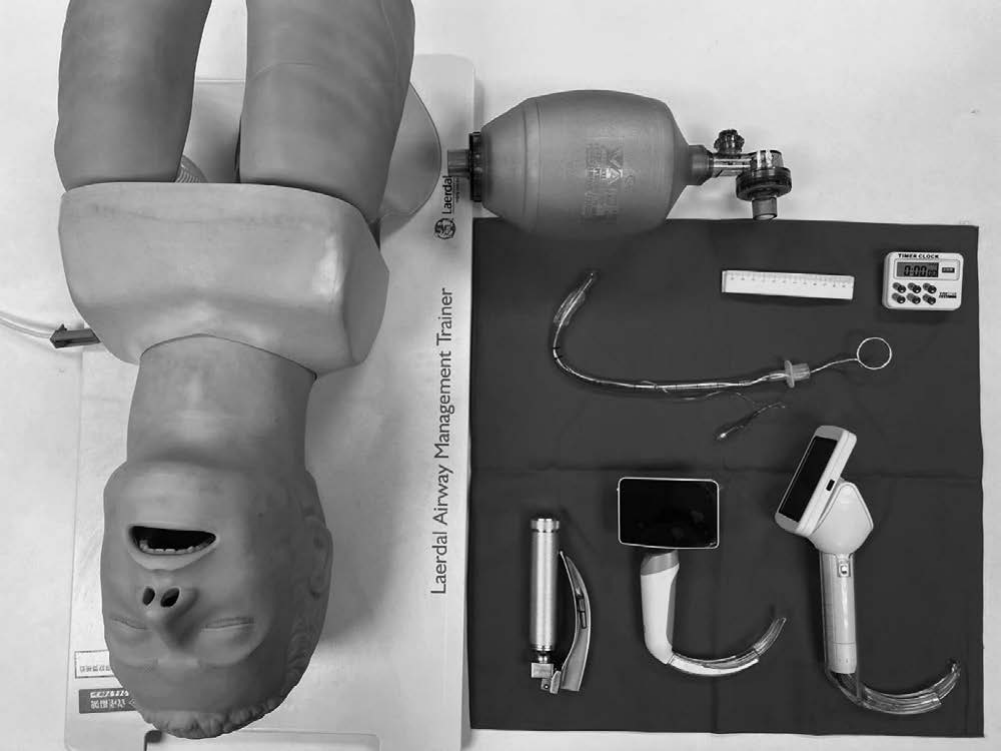
Intubation equipment.

This is a cross-sectional study. We designed a questionnaire to assess the perception of an operator performing intubation on a dummy model using the new partly disposable video laryngoscope (LA10000) versus another video laryngoscope and a conventional laryngoscope (Fig. 1). The questionnaire consisted of 3 parts: collecting basic information, conducting a satisfaction survey, and evaluating techniques. The operator was asked about the quality of the laryngoscope and its feel, maneuverability, ease of use, and video quality during laryngoscopy. Other metrics recorded were the time to visualization of the glottis, intubation time, intubation success rate, distance between the operator and the training model, and time from glottis visualization to tube insertion. In addition, a simple operation video was produced. This video mainly shows intubation using a video laryngoscope.

The participants in this study were 50 clinical staff members from 2 hospitals who met the inclusion criteria: having performed intubation or learned intubation techniques and volunteered to participate. The exclusion criteria were those who had not learned intubation techniques or did not know how to perform intubation. The participants were asked to perform tracheal intubation by inserting an endotracheal tube with a 7.5-mm internal diameter through the vocal cords, applying either a conventional laryngoscope with a size 3 blade or a video laryngoscope with a size 2 blade. Each participant used each device for a total of 3 intubation attempts. Participation in this study was voluntary. The researcher provided a Laerdal adult airway management training model and 3 laryngoscopes and explained the purpose of the research. The staff from one of the participating hospitals did not watch the simple operation video that we produced before they used the equipment. The 3 laryngoscope models used were a conventional Macintosh laryngoscope, Medcaptain VS-10s video laryngoscope, and Honestmc video laryngoscope_LA10000.

### 2.2. Ethics statement

This study was approved by the institutional review board of the Chang Gung Medical Foundation (202101175B1). Participation was voluntary, and written informed consent was obtained from all participants.

### 2.3. Statistical methods

We conducted a correlation analysis of operators’ basic data. All statistical data were analyzed using SPSS version 19.0. Basic data were analyzed using *t* tests or chi-square tests. Categorical variables were expressed as frequency distributions and percentages. Continuous variables were expressed as mean ± standard deviation or median, and multiple continuous variables were analyzed using analysis of variance. Statistical significance was set at *P* < .05 indicated significance.

The primary outcomes were time to glottic visualization, intubation time, intubation success rate, distance between the operator and training model, and time from glottic visualization to tube insertion. The secondary outcomes were overall laryngoscopic quality, operative feel, maneuverability, ease of use, and video quality.

## 3. Results

The study included 50 clinical participants who each completed intubation with 3 different devices with no missing data. Nineteen participants did not watch the operation video: 57.9% were from respiratory therapy, 42.1% from intensive care, 68.4% had no intubation experience, and 73.7% had no video laryngoscope experience. Of the 31 participants who watched the video: 35% worked in internal medicine, 48.4% in pediatrics, 41.9% had 1 to 3 years of intubation experience, and 77.4% had no video laryngoscope experience (Table 1).

**Table 1:** Basic information of participants.

	Have not seen the video operation videos	Have seen the video operation videos
Number	19	31
Age (yr; mean ± SD)	40.00 ± 12.58	31.77 ± 7.34
Sex male/female (n,%)	6 (31.6%)/13 (68.4%)	20 (64.5%)/11 (35.5%)
Department/Section (n,%)	ICU -8 (42.1%) Respiratory Therapy -11 (57.9%)	Internal Medicine -11 (35%) Intern -2 (6.5%) Pediatrics -15 (48.4%) Respiratory Therapy -2 (6.5%) Surgical -1 (3.2%)
Practical intubation experience (n,%)	No: 13 (68.4%) 1–3 yr: 1 (5.3%) 5–10 yr: 1 (5.3%) >10 yr: 4 (21.1%)	No: 4 (12.9%) <1 yr: 3 (9.7%) 1–3 yr: 13 (41.9%) 3–5 yr: 7 (22.6%) 5–10 yr: 2 (6.5%) >10 yr: 2 (6.5%)
Experience with video laryngoscope (n,%)	No: 14 (73.7%) Yes: 5 (26.3%)	No: 24 (77.4%) Yes: 7 (22.6%)

ICU = intensive care unit.

### 3.1. Satisfaction survey

Video laryngoscopes were superior to conventional laryngoscopes in terms of quality, operative feel, and ease of use (Table 2). However, regarding maneuverability, laryngoscopes were rated from best to worst as follows: Medcaptain VS-10s, Macintosh, and LA10000. If focusing on participants with extensive intubation experience, the results and order were similar. There was a difference only in laryngoscope quality, with LA10000 being superior to Medcaptain VS-10s and Macintosh.

**Table 2:** Compare satisfaction* with disposable video laryngoscopes and conventional laryngoscope.

	Have not seen the video operation videos (N = 19)			Have seen the video operation videos (N = 31)			Total of participants (N = 50)		
	Conventional laryngoscope	Disposable video laryngoscope (Medcaptain VS-10s)	Disposable video laryngoscope (LA10000)	Conventional laryngoscope	Disposable video laryngoscope (Medcaptain VS-10s)	Disposable video laryngoscope (LA10000)	Conventional laryngoscope	Disposable video laryngoscope (Medcaptain VS-10s)	Disposable video laryngoscope (LA10000)
Laryngoscope quality	3.42	4.05	4.11	3.68	4.23	4.03	3.58	4.16	4.06
Operative feel	3.58	4.16	3.74	3.68	4.16	3.74	3.64	4.16	3.74
Maneuverability	3.42	4.11	3.68	3.87	4.10	3.39	3.70	4.10	3.50
Ease of use	3.47	4.05	4.11	3.77	4.16	3.77	3.66	4.12	3.90
Video quality	Nil	4.05	4.42	Nil	4.29	4.26	Nil	4.20	4.32

* Satisfaction level in order: very satisfied 5 points, satisfied 4 points, fair 3 points, dissatisfied 2 points, very dissatisfied 1 point.

### 3.2. Evaluation of performance techniques

**Figure 2.**
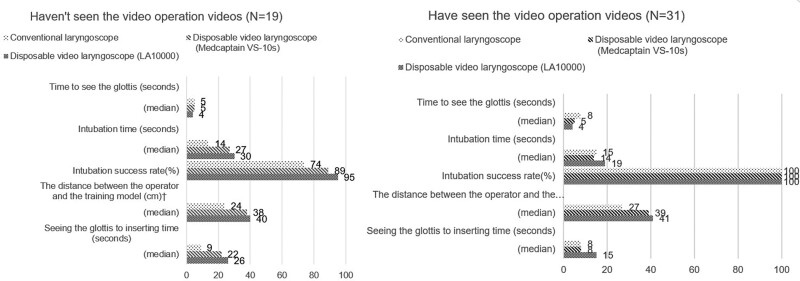
Evaluation of 3 laryngoscope intubation techniques.

The following operational technical assessment results were obtained (Table 3): The fastest time to glottis visualization was achieved using the LA10000, and this time differed significantly from that for the conventional method (4.76 ± 2.50 vs 8.08 ± 5.01 seconds, *P* < .05). Use of the LA10000 resulted in a longer intubation time than did use of the conventional method (25.69 ± 20.76 vs 19.66 ± 12.80 seconds, *P* < .05). The intubation success rate was higher when using LA10000 than when using the other methods (98% vs 96% vs 90%). The distance between the operator and training model was greatest when using the LA10000, and this distance differing significantly from that for the conventional method (41.25 ± 7.44 cm vs 27.50 ± 8.28 cm, *P* < .05). A greater distance reduced the operator risk of infection. The time from glottis visualization to tube insertion was longer for LA10000. However, if the operator had watched the operation video before performing the procedure, this time was significantly reduced (18.16 ± 12.65 vs 31.68 ± 28.36 seconds, *P* < .05; Fig. 2).

**Table 3:** Evaluation of 3 laryngoscope intubation techniques.

	Have not seen the video operation videos (N = 19)			Have seen the video operation videos (N = 31)			Total of participants (N = 50)		
	Conventional laryngoscope	Disposable video laryngoscope (Medcaptain VS-10s)	Disposable video laryngoscope (LA10000)	Conventional laryngoscope	Disposable video laryngoscope (Medcaptain VS-10s)	Disposable video laryngoscope (LA10000)	Conventional laryngoscope	Disposable video laryngoscope (Medcaptain VS-10s)	Disposable video laryngoscope (LA10000)
Time to see the glottis (seconds)									
(mean ± SD)	6.89 ± 5.32	6.37 ± 3.15	4.53 ± 2.41	8.81 ± 4.76	5.55 ± 2.10*	4.9 ± 2.57*	8.08 ± 5.01	5.86 ± 2.55*	4.76 ± 2.50*
(median)	5	5	4	8	5	4	7	5	4
Intubation time (seconds)									
(mean ± SD)	22.37 ± 17.98	35.05 ± 23.44*	36.21 ± 28.10*	18 ± 8.14	15.71 ± 6.73**	23.06 ± 12.73**	19.66 ± 12.80	23.06 ± 17.87*	25.69 ± 20.76*
(median)	14	27	30	15	14	19	15	18	25.50
Intubation success rate (%)	74	89	95	100	100	100	90	96	98
The distance between the operator and the training model (cm)†									
(mean ± SD)	27.53 ± 10.19	38.03 ± 6.54*	40.34 ± 6.77*	27.48 ± 7.05	39.42 ± 5.75*	41.81 ± 7.88*	27.50 ± 8.28	38.89 ± 6.03	41.25 ± 7.44*
(median)	24	38	40	27	39	41	26	38.25	41
Seeing the glottis to inserting time (seconds)									
(mean ± SD)	15.47 ± 14.92	28.68 ± 24.71*	31.68 ± 28.36*	9.19 ± 5.08	10.16 ± 6.06**	18.16 ± 12.65*/**	11.58 ± 10.35	17.20 ± 18.15*	20.78 ± 20.91*
(median)	9	22	26	8	8	15	8	11	16

* Compare with conventional laryngoscope (*P* < .05).

**“ seen the video operation videos” is faster than “haven’t seen” (*P* < .05).

† The distance between the operator and the training model is measured from the corner of the operator’s mouth to the corner of the manikin mouth.

## 4. Discussion

In this study, video laryngoscopes were superior to conventional laryngoscopes in terms of quality, operative feel, and ease of use. The LA10000 video laryngoscope resulted in the shortest glottis visualization time, but intubation took longer than with the other methods. Similarly, Szarpak et al (2017) found that the median time to intubation was 29.5 seconds when a conventional Macintosh laryngoscope was used but 229 seconds when an Intubrite video laryngoscope was employed.^[10]^ Cordovani et al (2019) compared the Macintosh direct laryngoscope with the GlideScope video laryngoscope. The video laryngoscope provided a superior glottic view despite requiring a lower median peak and average forces; however, the average time required to position the endotracheal tube at the laryngeal inlet was greater.^[11]^ Varsha et al (2019) found that the median time to intubation was longer when an Airtraq video laryngoscope was used than when a classic Macintosh laryngoscope was employed (13 seconds vs 11 seconds, *P* = .05).^[12]^ Thus, video laryngoscopes provide a superior glottic view and result in higher intubation success rates compared to conventional laryngoscopes, but intubation may take longer when they are used.^[10–12]^ In our study, the median time to intubation was 25.5 seconds for the LA10000 video laryngoscope, 18 seconds for the Medcaptain VS-10s video laryngoscope, and 15 seconds for the conventional laryngoscope.

**Figure 3.**
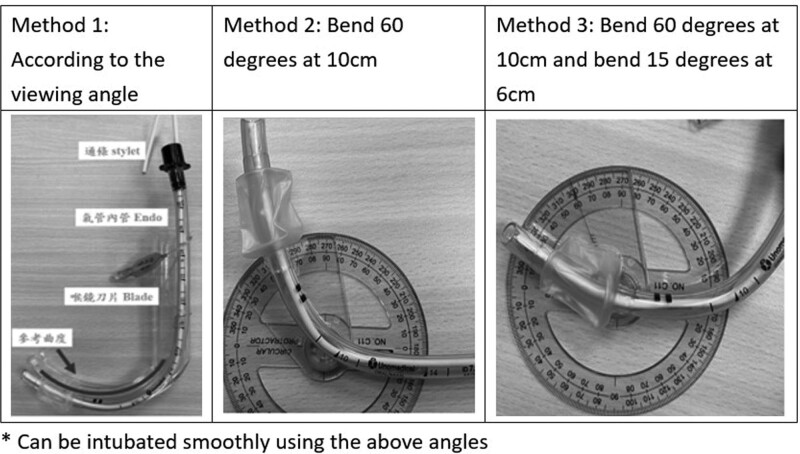
Endotracheal tube angle schema.

We identified that the angle of the endotracheal tube was crucial when intubating with a video laryngoscope, and a simple video of the operation and the endotracheal tube angle schema were created (Fig. 3). We suggest that 10 cm from the front end of the endotracheal tube, a 60° angle should be made^[13]^ or the endotracheal tube should be bent by 60° at a point 10 cm from its distal end and by an additional 10° at a point 6 cm from the distal end. Similarly, Lee et al (2018) demonstrated that stylet angulation of 70° reduced the time to intubation when a GlideScope video laryngoscope was used.^[14]^ In their study, they compared 2 GlideScope stylet angulations (90° vs 70°) in terms of time to intubation. An inappropriate stylet angle or shape may lead to failed intubation, causing airway trauma and edema. Finally, we identified that if the operator had seen the video before executing the procedure, the glottis visualization to tube insertion time was significantly shorter. However, the intubation success rate and distance between the operator and the training model were greatest when LA10000 was used, indicating a reduced risk of operator infection. Video laryngoscopy significantly enhances infection prevention measures, especially during the COVID-19 pandemic. Like studies indicate that it improves visualization of anatomical structures, speeds up intubation procedures, and increases first-attempt success rates, thereby reducing the risk of aerosol generation.^[15–17]^ The present findings are consistent with the results of other studies^[6,9,18,19]^ and indicate that video laryngoscopy results in superior laryngeal views and higher intubation success rates than conventional laryngoscopy.

The advantages of video laryngoscopes over conventional laryngoscopes include improved visualization of the larynx, higher intubation success rate, possibly higher patient comfort, and lower risk of trauma to the larynx. A greater distance between the corners of the operator and the patient mouth was also determined. The disadvantages of video laryngoscopes include greater costs, greater time to intubation, and potential technical difficulties. By contrast, conventional intubation techniques are less costly and lead to shorter intubation times, and operators are more familiar with them. The disadvantages of conventional intubation techniques include poor laryngeal visualization, a greater risk of laryngeal trauma, and lower intubation success rates. However, the current role of video laryngoscopy involves its widespread adoption in various medical settings, particularly in airway management during intubation procedures. It offers advantages such as improved visualization of the airway anatomy, enhanced teaching capabilities, and potentially higher intubation success rates, especially in difficult airway situations. Additionally, video laryngoscopy is increasingly integrated into medical education and training programs to improve intubation skills among healthcare professionals. Its versatility and effectiveness have positioned it as a valuable tool in modern airway management practices. Like Gómez-Ríos et al mention it is recommended to use it universally as the primary device for all tracheal intubations^[2]^ (Guideline for difficult airway management).

Our study had several limitations. First, the operators who performed the intubations using the tested devices were not fully blinded to the device used, which may have introduced a bias in the study variables. Each participant used each device for a total of 3 intubation attempts; thus, their first attempt may have affected their performance in subsequent attempts. Second, our study was conducted using a training model rather than actual patients; therefore, the results may not fully reflect the performance of these devices in clinical practice. Further studies are required to validate our findings in real-world scenarios.

## 5. Conclusion

The results of this study indicate that video laryngoscopes, especially the partially disposable LA10000, are superior to conventional laryngoscopes in terms of quality, operative experience, and ease of use. When LA10000 was employed, the intubation success rate was higher, and the operator risk of infection was lower because of the greater distance from the training model. However, the use of LA10000 led to longer intubation and glottic visualization of tube insertion times. This study also demonstrated that when participants viewed a simple operation video prior to using LA10000, the tube insertion time was shorter. These findings indicated that video laryngoscopes are safer and more effective than conventional laryngoscopes, particularly during the COVID-19 pandemic. However, further education and training should be provided to ensure proper use of these devices. In addition, future studies should investigate the long-term cost-effectiveness and clinical outcomes associated with the use of video laryngoscopes.

## Author contributions

**Data curation:** Hui-Chin Chen, Jui-Fang Liu, Miao-Ching Chi, Hsiu-Lan Cheng.

**Formal analysis:** Hui-Chin Chen.

**Investigation:** Hui-Chin Chen, Jui-Fang Liu, Hsiu-Lan Cheng.

**Supervision:** Miao-Ching Chi.

**Writing – original draft:** Hui-Chin Chen.

**Writing – review & editing:** Hui-Chin Chen, Jui-Fang Liu.
